# Comparative Study of an Antioxidant Compound and Ethoxyquin on Feed Oxidative Stability and on Performance, Antioxidant Capacity, and Intestinal Health in Starter Broiler Chickens

**DOI:** 10.3390/antiox13101229

**Published:** 2024-10-13

**Authors:** Yong Xiao, Xuyang Gao, Jianmin Yuan

**Affiliations:** State Key Laboratory of Animal Nutrition and Feeding, College of Animal Science and Technology, China Agricultural University, Beijing 100193, China; xiaoyong_hengyang@cau.edu.cn (Y.X.); xuyang2019@yeah.net (X.G.)

**Keywords:** antioxidant compound, ethoxyquin, broiler, antioxidant capacity, intestinal health

## Abstract

Concerns over the safety of ethoxyquin (EQ) highlight the need for safer, more effective feed antioxidants. This study investigated a healthier antioxidant compound (AC) as a potential alternative to EQ in broilers. A total of 351 one-day-old Arbor Acres Plus male broilers were randomly assigned to three treatments for 21 days: control (CON), EQ group (200 g/ton EQ at 60% purity), and AC group (200 g/ton AC containing 18% butylated hydroxytoluene, 3% citric acid, and 1% tertiary butylhydroquinone). AC supplementation reduced the acid value, peroxide value, and malondialdehyde content in stored feed, decreased feed intake and the feed conversion ratio without affecting body weight gain, and enhanced antioxidant capacity (liver total antioxidant capacity and superoxide dismutase; intestinal catalase and glutathione peroxidase 7). It improved intestinal morphology and decreased barrier permeability (lower diamine oxidase and D-lactate), potentially by promoting ZO-1, Occludin, and Mucin2 expression. The AC also upregulated NF-κB p50 and its inhibitor (NF-κB p105), enhancing immune regulation. Additionally, the AC tended to increase beneficial gut microbiota, including *Lactobacillus*, and reduced *Bacteroides*, *Corprococcus*, and *Anaeroplasma*. Compared to EQ, the AC further enhanced feed oxidative stability, the feed conversion ratio, intestinal morphology and barrier functions, and inflammatory status, suggesting its potential as a superior alternative to EQ for broiler diets.

## 1. Introduction

Animal diets rich in lipids, particularly polyunsaturated fatty acids, are prone to oxidation. This oxidation process can be exacerbated by factors such as elevated temperatures, light exposure, and metal ions during feed production [1]. The oxidation of feed lipids not only diminishes the nutritional value of the feed [2] but also adversely affects the normal physiological functions of the animal [3,4]. The ingestion of oxidized diets can impair the intestinal villus morphology and barrier function, reduce antioxidant capacity, promote intestinal inflammation, and disturb the intestinal microbiota, ultimately impacting the production performance of animals [5,6]. Incorporating antioxidants into feed to mitigate oxidation is an effective strategy. Ethoxyquin (EQ) is the most widely used antioxidant in feed due to its low cost and high antioxidant capacity [7]. However, concerns regarding the safety of ethoxyquin and its metabolites have been raised [8]. Recent studies have further reported that ethoxyquin and its metabolites can remain in various tissues of animals, which may pose a potential hazard for consumers [9,10]. Consequently, there is an urgent need to identify safer and more effective antioxidant alternatives.

Butylated hydroxytoluene (BHT) is another widely used antioxidant [11]. Recent studies have demonstrated its favorable safety profile in broilers and *Micropterus salmoides* even when administered at ten times the maximum allowable additive dose (1500 mg/kg) [12,13]. Tertiary butylhydroquinone (TBHQ) is another antioxidant used for antioxidant protection in food, with evidence suggesting that its antioxidant capacity surpasses that of BHT [14]; however, its higher cost limits its applicability. Recent research highlights the potential synergistic effects of combining antioxidants with different properties [15,16]. In vitro studies have shown a synergistic effect between BHT and TBHQ in stabilizing soybean biodiesel [17]. Additionally, citric acid is frequently added to feed as a synergist to chelate metal ions from containers and the environment, thereby mitigating their pro-oxidative effects [18,19]. Studies indicate that citric acid and TBHQ exhibit a strong synergistic effect, significantly reducing the peroxide value of peanuts during storage [20]. Given the synergistic effects of various antioxidants and the metal-chelating properties of citric acid, a novel antioxidant compound (AC) comprising BHT, TBHQ, and citric acid may provide superior protection compared to ethoxyquin. Current research on antioxidant combinations primarily focuses on in vitro studies, with limited application in animal diets. This study aims to assess the effects of incorporating a blend of BHT, TBHQ, and citric acid into broiler diets, evaluating its antioxidant activity in feed, impact on broiler performance, antioxidant capacity, and impact on intestinal health and comparing its efficacy with that of ethoxyquin.

## 2. Materials and Methods

### 2.1. Animals and Experimental Design

All animal procedures in this experiment were approved by the Institutional Animal Care and Use Committee of China Agricultural University, Beijing, China (Permit Number: AW52704202-1-1). A total of 351 one-day-old Arbor Acres Plus male broilers were randomly assigned to three groups, each consisting of nine replicates with 13 chickens per replicate. The treatments included a CON group (basal diet), an EQ group (basal diet supplemented with 200 g/ton of EQ at 60% purity, effective content 120 g/ton), and an AC group (basal diet supplemented with 200 g/ton of AC, containing 18% BHT, effective content 36 g/ton; 3% citric acid, effective content 6 g/ton; and 1% TBHQ, effective content 2 g/ton). EQ, BHT, citric acid, and TBHQ were purchased from Beijing Sunpu Biochemical and Technical Co., Ltd. (Beijing, China). The experiment lasted 21 days. Feed was provided in crumble form, and the nutritional composition of the basal diet is presented in Table 1.

All broilers were raised in a fully enclosed, windowless poultry house utilizing a caged system, ensuring controlled environmental conditions. The cages measured 100 cm in length, 70 cm in width, and 40 cm in height, resulting in a stocking density of 18.6 birds/m^2^. Birds had ad libitum access to feed and water throughout the entire feeding period. Room temperature was maintained at 33 °C during the first four days of the study and was gradually decreased to 23 °C by the end of the third week. Relative humidity was maintained at 65–70% from days 1 to 7 and at 50–65% from days 8 to 21. Illumination was provided by incandescent lamps, with a light intensity ranging from 10 to 20 lux. The lighting schedule consisted of 24 h of light from days 1 to 2, 23 h on day 3, 22 h on day 4, 21 h on day 5, and 20 h from days 6 to 21. Animal management practices adhered to standards established for Arbor Acres Plus broilers.

### 2.2. Feed Oxidation Stability Testing

After storing the feed for three or six weeks, oil samples were extracted to test the oxidation stability index. The specific procedures are as follows: Following the completion of feed preparation, 1.2 kg of each feed was allocated to three replicates situated at distinct locations within the chicken house. During the 3rd and 6th weeks, 200 g of feed was sampled, ground into a powder, passed through a 40-mesh screen, and stored at −20 °C. Oil was then extracted to identify indicators associated with oxidation stability. The oil extraction process involved taking a suitably weighted sample and placing it in a wide-mouth bottle. Approximately four times the sample’s weight in petroleum ether was added and the mixture was stirred with a glass rod before tightly sealing the bottle. The bottle was then shaken on a reciprocating oscillator for two hours. Subsequently, the solution was transferred to a 50 mL centrifuge tube and centrifuged at 8000 r/min for five minutes. The supernatant was collected and transferred to a rotary evaporator with the water bath temperature maintained below 40 °C. The solvent was evaporated under reduced pressure, yielding the remaining liquid oil as the final sample. The determination of the acid value (AV) and peroxide value (POV) was carried out according to Ma et al. [21]. Malondialdehyde (MDA) content was measured using a commercial kit (#BC0025, Beijing Solarbio Biotech, Beijing, China).

### 2.3. Assessment of Growth Performance

The body weight of broilers on days 1 and 21, along with feed input on day 1 and the remaining feed weight on day 21, were measured using an electronic platform scale (Wuxi Yingheng Electronic Technology Co., Ltd., Wuxi, China) with a maximum capacity of 60 kg and a measurement accuracy of 1 g. The average body weight of the chickens per cage was calculated by dividing the total weight of the chickens in each cage by the number of chickens. The average body weight gain (BWG) per cage was calculated by subtracting the average body weight on day 1 from that on day 21. The feed conversion ratio (FCR) was calculated by dividing the total feed consumption by the total body weight gain in each cage, including the weight of any dead or culled birds. Feed intake (FI) was adjusted by subtracting the feed consumption of dead or culled birds (calculated as the product of their body weight gain and FCR) from the total feed consumption of each cage and then dividing the result by the number of remaining birds.

### 2.4. Sample Collection

At 21 days of age, a healthy chick close to the average weight was selected from each replicate, electrically stunned, and then bled via the carotid artery. A 4 mL blood sample was collected in a coagulation tube. After centrifugation at 3000× *g* for 10 min at 4 °C, the serum was separated and stored at −20 °C. The small intestine was removed, and approximately 1 cm sections from the middle regions of the duodenum and jejunum were collected and fixed in a 4% paraformaldehyde solution for subsequent intestinal morphology measurements. Additionally, liver tissue, jejunal tissue, and cecal contents were collected and immediately frozen in liquid nitrogen and stored at −80 °C until further analysis.

### 2.5. Measurement of Liver Antioxidant Capacity

The activities of total antioxidant capacity (T-AOC, #A015-2), superoxide dismutase (SOD), catalase (CAT, #A007-1), glutathione peroxidase (GSH-Px, #A005-1), glutathione (GSH, #A006-2), and malondialdehyde (MDA, #A003-1) in the liver were evaluated and normalized to total protein content (BCA, #A045-4) using kits from Nanjing Jiancheng Bioengineering Institute (Nanjing, China).

### 2.6. Intestinal Morphology Analysis

Tissue sections were fixed in 4% paraformaldehyde, embedded in paraffin, sectioned, dehydrated, and stained with hematoxylin and eosin by Sevier Biotechnology Co., Ltd. (Wuhan, China). Villus height (VH) and crypt depth (CD) were measured using a Leica DM 750 optical microscope (Leica Microsystems, Wetzlar, Germany), and the ratio of villus height to crypt depth (VCR) was calculated from these measurements.

### 2.7. Measurement of Intestinal Barrier Permeability

Diamine oxidase (DAO) activity (#BC1285) and D-lactate content (#BC5355) in serum were measured according to protocols provided by kits from Beijing Solarbio Biotech (Beijing, China).

### 2.8. Expression of Intestinal-Barrier-, Antioxidant-, and Inflammation-Related Proteins

Jejunal mucosa (100 mg) was homogenized in 1 mL of radioimmunoprecipitation assay buffer (Beijing Solarbio Biotech, Beijing, China) with 1 mM phenylmethylsulfonyl fluoride using a SCIENTZ-12 high-throughput tissue homogenizer (Xinzhi Biotech, Ningbo, China) followed by cell disruption with a tissue disruptor (Xinzhi Biotech, Ningbo, China). After incubation on ice for 30 min, the samples were centrifuged at 12,000× *g* for 15 min to collect the supernatant. Soluble proteins were separated by sodium dodecyl sulfate polyacrylamide gel electrophoresis (100 V, 2 h), transferred to a nitrocellulose membrane (120 V, 40 min), and subsequently blocked with 5% skimmed milk powder (Sangon Biotech, Shanghai, China). The nitrocellulose membrane was then probed with primary antibodies overnight at 4 °C, followed by incubation with secondary antibodies for 1 h at room temperature. Western blot bands were scanned and analyzed using a fully automated chemiluminescence imaging system, with band density normalized to β-actin content. The primary antibodies used were as follows: Occludin (#DF7504) and ZO-1 (#AF5154) from Affinity Bioscience (OH, USA); MUC2 (#sc-515032) and HO-1 (#sc-136960) from Santa Cruz (TX, USA); and KEAP1 (#10503-2-AP), NRF2 (#16396-1-AP), GPX7 (#13501-1-AP), NQO1 (#11451-1-AP), TLR4 (#19811-1-AP), IκB (#10268-1-AP), and NF-κB1 (p50 and p105) (#14220-1-AP) from Proteintech (Wuhan, China). β-actin (#I102) served as the internal reference protein (Bioworld Biotech, Nanjing, China). The secondary antibodies employed included anti-rabbit HRP secondary antibody (#7074) and anti-mouse HRP secondary antibody (#7076), both from Cell Signaling Technology (Shanghai, China).

### 2.9. Expression of Intestinal-Inflammation-Related Genes

Total RNA was extracted using RNAiso Plus (#9108, Takara, Japan) and transcribed into cDNA with the Prime Script™ RT Reagent Kit with gDNA Eraser (#RR047A, Takara, Japan). Real-time fluorescence quantitative PCR reactions were performed with the SYBR Premix Ex Taq kit (#RR420A, Takara, Japan) on an ABI7500 fluorescence quantitative PCR instrument (Applied Biosystems, CA, USA). All primer sequences for target genes are presented in Table 2. The relative expression levels of each gene were normalized to β-actin and analyzed using the 2^−ΔΔCt^ method.

### 2.10. Cecal Microbiota Analysis

Cecal chyme samples underwent high-throughput amplicon sequencing of the 16S rRNA gene V3-V4 region at Shanghai Paison Biotechnology Co., Ltd. (Shanghai, China), using the Illumina MiSeq platform (primer sequences: F: ACTCCTACGGGAGGCAGCA; R: GGACTACHVGGGTWTCTAAT). Raw sequencing data were denoised using the QIIME2 DADA2 analysis pipeline to obtain Amplicon Sequence Variants (ASVs) with 97% similarity. The ASVs were then annotated with the Greengenes 13.8 database. Based on the species annotation results, both alpha and beta diversity were calculated, and differences between groups were analyzed to elucidate the distinct characteristics of microbial community structures under varying treatments.

### 2.11. Statistical Analysis

A one-way ANOVA was conducted using SPSS 26.0 software, and multiple comparisons were performed using Duncan’s method. Prior to ANOVA, normality and homoscedasticity were assessed using Shapiro–Wilk and Levene tests, respectively. Percentages were compared after an arcsine transformation. The results are presented as mean ± standard error of the mean (SEM). Statistical significance was accepted at *p* < 0.05, while a statistical trend was considered when 0.05 ≤ *p* < 0.10. Graphs were generated using GraphPad Prism 10.

## 3. Results

### 3.1. Oxidative Stability of Feeds

**Figure 1:**
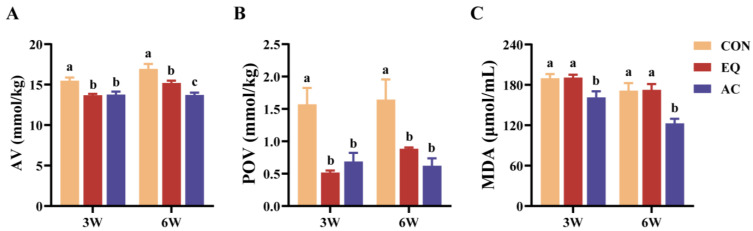
Oxidative stability of feeds after three and six weeks of storage. N = 3. (**A**) Acid value (AV); (**B**) peroxide value (POV); (**C**) malondialdehyde (MDA) content. CON, basal diet; EQ, basal diet + 200 g/ton ethoxyquin; AC, basal diet + 200 g/ton antioxidant compound. ^abc^ Different superscript letters indicate significant differences (*p* < 0.05).

Dietary supplementation with EQ or the AC significantly decreased the AV and POV of feeds after three and six weeks of storage (*p* < 0.05) (Figure 1A,B). Notably, the AC group exhibited a lower AV than the EQ group at the sixth week (*p* < 0.05). Furthermore, supplementation with the AC significantly reduced the MDA content in the diets at both the third and sixth weeks compared to the CON and EQ groups (*p* < 0.05) (Figure 1C).

### 3.2. Growth Performance

**Figure 2:**
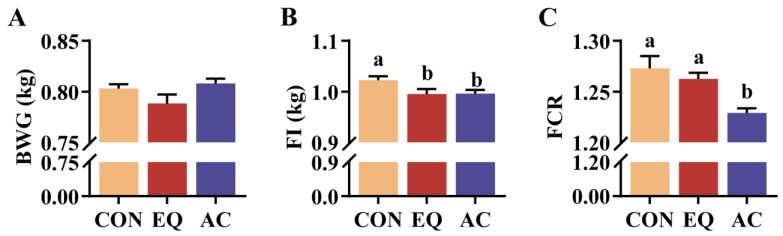
Growth performance of broilers. N = 9. (**A**) Body weight gain (BWG); (**B**) feed intake (FI); (**C**) feed conversion ratio (FCR). CON, basal diet; EQ, basal diet + 200 g/ton ethoxyquin; AC, basal diet + 200 g/ton antioxidant compound. ^ab^ Different superscript letters indicate significant differences (*p* < 0.05).

The inclusion of the AC in the diet tended to increase the BWG compared to the EQ group (0.05 ≤ *p* < 0.10) (Figure 2A); however, no significant difference was observed between the EQ and CON groups. Additionally, both AC and EQ supplementation significantly decreased FI in broilers (*p* < 0.05) (Figure 2B). Furthermore, supplementation with the AC significantly reduced the FCR compared to both the CON and EQ groups (*p* < 0.05), with no significant difference observed between the latter two groups (Figure 2C).

### 3.3. Liver Antioxidant Capacity

**Figure 3:**
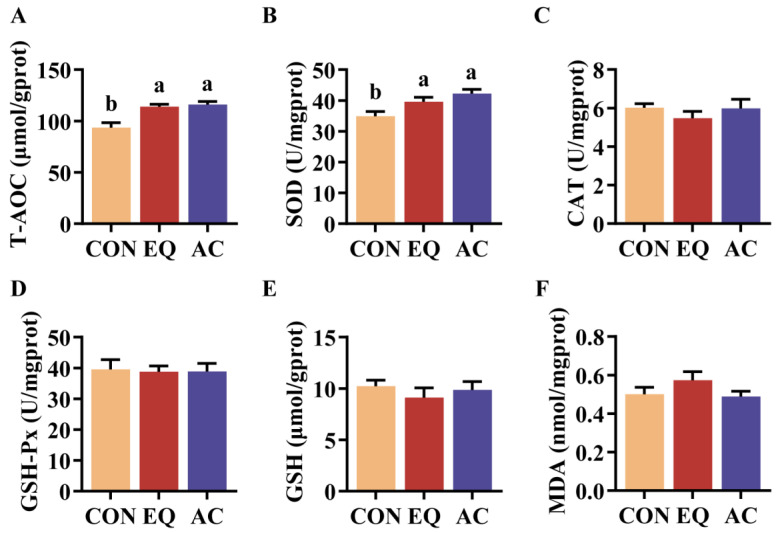
Liver antioxidant capacity of broilers. N = 8. (**A**) Total antioxidant capacity (T-AOC); (**B**) superoxide (SOD) activity; (**C**) catalase (CAT) activity; (**D**) glutathione peroxidase (GSH-Px) activity; (**E**) reduced glutathione (GSH) content; (**F**) malondialdehyde (MDA) content. CON, basal diet; EQ, basal diet + 200 g/ton ethoxyquin; AC, basal diet + 200 g/ton antioxidant compound. ^ab^ Different superscript letters indicate significant differences (*p* < 0.05).

Both AC and EQ dietary supplementation significantly increased T-AOC and SOD activities in the livers of broilers compared to the CON group (*p* < 0.05) (Figure 3A,B). However, no significant differences were found in the levels of CAT, GSH-Px, GSH, and MDA among the groups (Figure 3C–F).

### 3.4. Intestinal Morphology

In comparison to the CON group, dietary supplementation with EQ tended to decrease duodenal VH (0.05 ≤ *p* < 0.10) (Table 3). Conversely, the VCR in the duodenum was significantly higher in the AC group than in the other groups (*p* < 0.05). Furthermore, the jejunal VH in the EQ group was significantly lower than that in the CON group (*p* < 0.05), while both the jejunal VH and CD in the AC group were significantly higher than those in both the CON and EQ groups (*p* < 0.05). Additionally, compared to the EQ group, the VCR in the jejunum was significantly higher in both the AC and CON groups (*p* < 0.05).

### 3.5. Intestinal Barrier Permeability and Expression of Related Proteins

**Figure 4:**
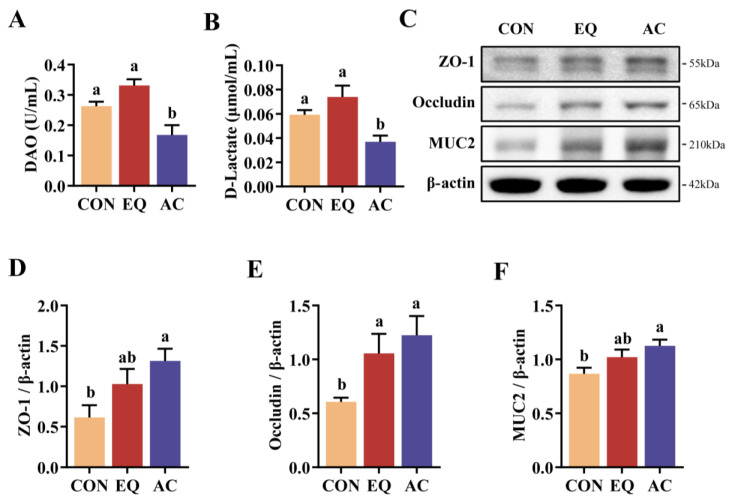
Intestinal barrier permeability and intestinal-barrier-related protein expression. N = 8. (**A**) Serum diamine oxidase activity and (**B**) D-lactate contents. (**C**–**F**) The relative protein expression of ZO-1, Occludin, and MUC2 in the jejunum. CON, basal diet; EQ, basal diet + 200 g/ton ethoxyquin; AC, basal diet + 200 g/ton antioxidant compound. Abbreviations: DAO, diamine oxidase. ^ab^ Different superscript letters indicate significant differences (*p* < 0.05).

AC supplementation significantly reduced serum DAO activity and D-lactate levels compared to both the CON and EQ groups (*p* < 0.05) (Figure 4A,B). Moreover, the AC significantly increased the relative protein expression of ZO-1, Occludin, and MUC2 (*p* < 0.05) (Figure 4C–F). EQ supplementation also significantly elevated Occludin expression (*p* < 0.05) (Figure 4C,E). While there was a trend towards higher expression of ZO-1 and MUC2 in the EQ group compared to the CON group, these differences were not statistically significant (*p* > 0.05) (Figure 4C,D,F).

### 3.6. Intestinal Antioxidant Capacity

**Figure 5:**
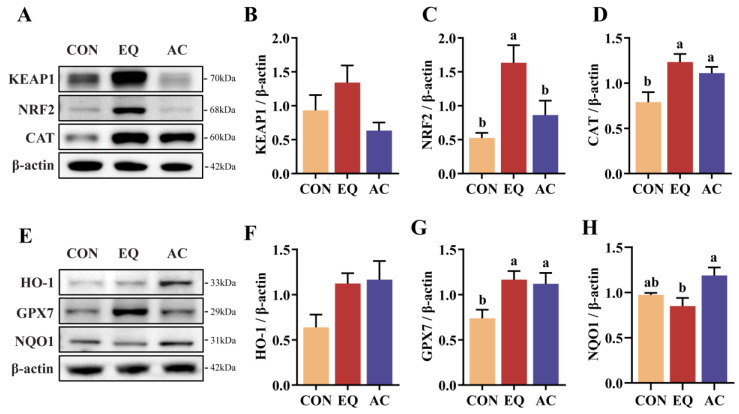
Intestinal-antioxidant-related protein expression. N = 8. (**A**–**D**) Relative protein levels of kelch-like ECH-associated protein 1 (KEAP1), nuclear factor erythroid 2-related factor 2 (NRF2), and catalase (CAT) in jejunum. (**E**–**H**) Relative protein levels of heme oxygenase-1 (HO-1), glutathione peroxidase 7 (GPX7), and NAD(P)H quinone dehydrogenase 1 (NQO1) in jejunum. N = 8. CON, basal diet; CON, basal diet; EQ, basal diet + 200 g/ton ethoxyquin; AC, basal diet + 200 g/ton antioxidant compound. ^ab^ Different superscript letters indicate significant differences (*p* < 0.05).

Dietary AC supplementation showed a tendency to reduce KEAP1 protein expression compared to the EQ group (0.05 ≤ *p* < 0.10), while NRF2 protein expression was significantly higher in the EQ group than in both the CON and AC groups (*p* < 0.05) (Figure 5A–C). Additionally, supplementation with both the AC and EQ significantly increased the relative expression levels of CAT and GPX7 compared to the CON group (*p* < 0.05), with a trend toward higher HO-1 expression (0.05 ≤ *p* < 0.10) (Figure 5D–G). Notably, NQO1 protein expression was significantly higher in the AC group than in the EQ group (*p* < 0.05), whereas no significant difference was observed between the CON and EQ groups (*p* > 0.05) (Figure 5E,H).

### 3.7. Expression of Intestinal-Inflammation-Related Cytokines

**Figure 6:**
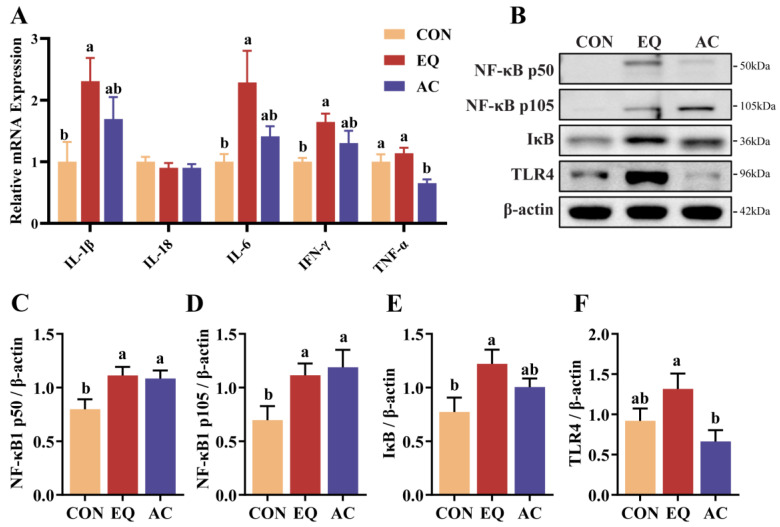
Expression of inflammatory cytokines at gene level and protein level. N = 8. (**A**) Relative mRNA expression of *IL-1β*, *IL-18*, *IL-6*, *IFN-γ*, and *TNF-α* in jejunum. (**B**–**F**) Relative protein levels of NF-κB p50, NF-κB p105, inhibitor of nuclear factor kappa-B (IκB), and toll-like receptor 4 (TLR4) in jejunum. CON, basal diet; EQ, basal diet + 200 g/ton ethoxyquin; AC, basal diet + 200 g/ton antioxidant compound. ^ab^ Different superscript letters indicate significant differences (*p* < 0.05).

EQ supplementation significantly increased the mRNA expression levels of *IL-1β*, *IL-6*, and *IFN-γ* compared to the CON group (*p* < 0.05), while AC supplementation showed a tendency to increase the expression levels of these cytokines, though the differences were not statistically significant (*p* > 0.05) (Figure 6A). Additionally, AC supplementation significantly reduced *TNF-α* gene expression (*p* < 0.05). Both the EQ and AC diets significantly upregulated NF-κB p50 and NF-κB p105 compared to the CON group (*p* < 0.05) (Figure 6B–D). Furthermore, the EQ group exhibited significantly higher expression levels of IκB and TLR4 proteins compared to the CON and AC groups, respectively (*p* < 0.05) (Figure 6B,E,F).

### 3.8. Cecal Microbiota

#### 3.8.1. Microbial Diversity

**Figure 7:**
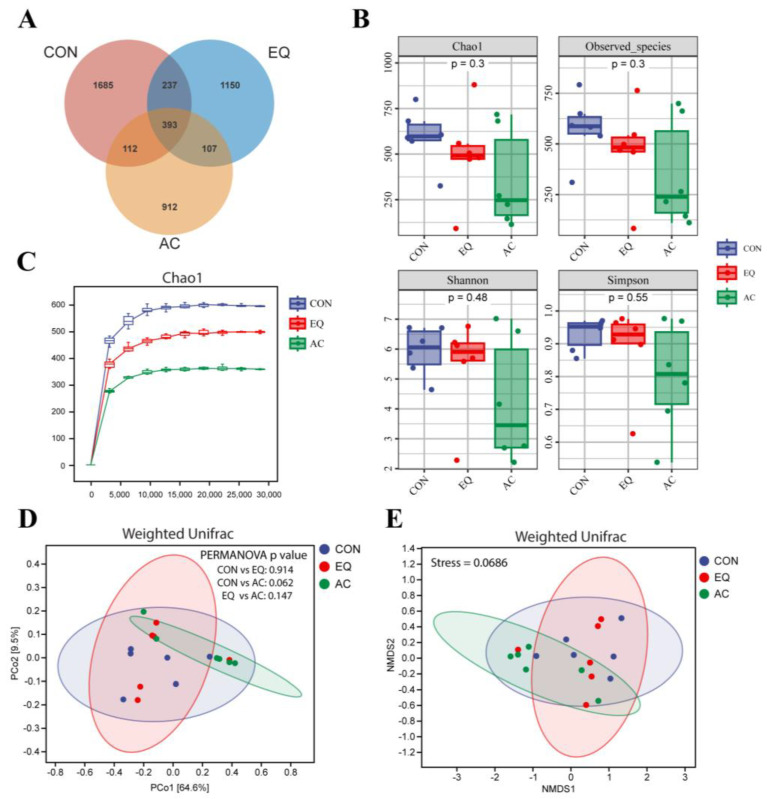
Changes in microbial diversity in the cecum of broilers. N = 6. (**A**) Venn diagram of the operational taxonomic units (OTUs) among all groups. (**B**) Bacterial richness (Chao1 and Observed species) and diversity (Shannon and Simpson) were evaluated by Kruskal–Wallis rank sum test and Dunn’s test. (**C**) The rarefaction curve shows the Chao1 index of each group under the same sampling depth. (**D**) The principal coordinate analysis (PCoA) and (**E**) non-metric multidimensional scaling (NMDS) analysis were conducted at the ASV level (the distance matrix was analyzed for statistical significance using PERMANOVA). CON, basal diet; EQ, basal diet + 200 g/ton ethoxyquin; AC, basal diet + 200 g/ton compound antioxidant.

The α and β diversity of intestinal microbiota in broilers under different treatments is presented in Figure 7. The Venn diagram demonstrates that the CON group, EQ group, and AC group contain 1685, 1150, and 912 unique ASVs, respectively, with 393 ASVs shared across all groups. Notably, the number of shared ASVs between the AC and CON groups, as well as between the AC and EQ groups, was 112 and 107, respectively, which is much lower than the number of common ASVs (237) observed between the EQ and CON groups (Figure 7A). No significant differences were found in the richness or diversity of cecal microbial communities among the groups (*p* > 0.05) (Figure 7B). The rarefaction curve based on the Chao1 index indicated that as the sampling depth increases, the curve begins to flatten, suggesting that sufficient reads have been obtained to represent each microbial community (Figure 7C). A principal coordinate analysis (PCoA) and non-metric multidimensional scaling (NMDS) were conducted using the same distance matrix, revealing a separation trend between the AC and CON groups (PERMANOVA, *p* = 0.062) (Figure 7D,E).

#### 3.8.2. Microbial Composition Analysis

**Figure 8:**
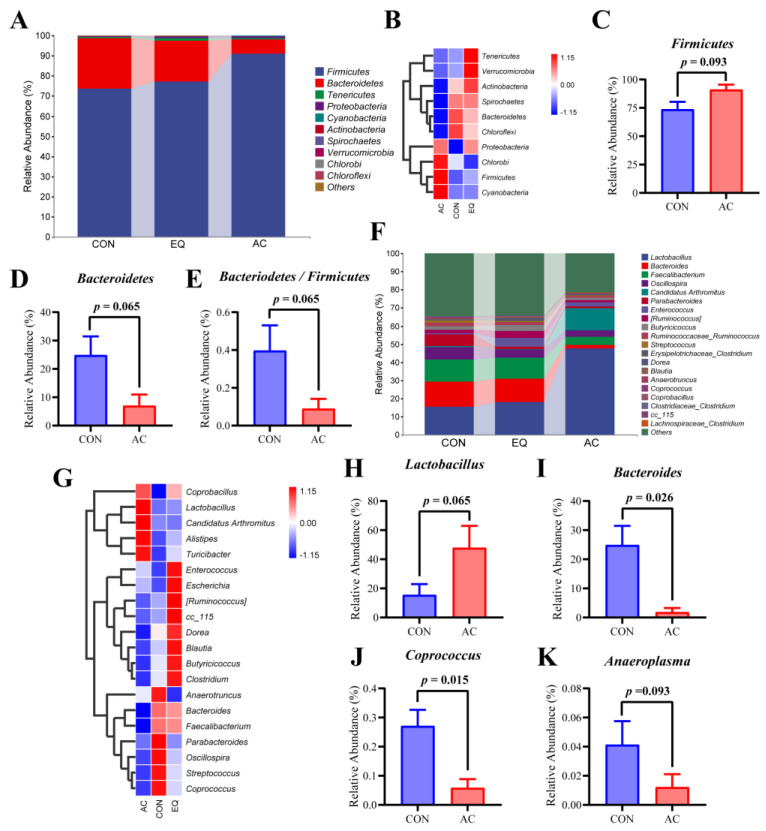
Changes in microbial community composition in the cecum of broilers. N = 6. (**A**) Distribution of cecal microbiota at the phylum level. (**B**) Heatmap showing the relative abundance of cecal microbiota at the phylum level. (**C**–**E**) Bacteria with differences in relative abundance among the top 10 phyla, including Firmicutes, Bacteroidetes, and the ratio of Bacteroidetes and Firmicutes. (**F**) Distribution of cecal microbiota at the genus level. (**G**) Heatmap showing the relative abundance of cecal microbiota at the genus level. (**H**–**K**) Bacteria with differences in relative abundance among the top 20 genera, including *Lactobacillus*, *Bacteroides*, *Coprococcus*, and *Anaeroplasma*. Differences in microbial abundance between the CON and AC groups were analyzed using the Wilcoxon rank sum test. CON, basal diet; EQ, basal diet + 200 g/ton ethoxyquin; AC, basal diet + 200 g/ton antioxidant compound.

The taxonomic composition of the top 10 phyla and top 20 genera in the cecal microbial communities of broilers from each group was analyzed (Figure 8). At the phylum level, Firmicutes and Bacteroidetes were identified as the dominant bacterial groups, with average relative abundances of 80.64% and 17.39%, respectively, in the cecal microbiota of AA broilers (Figure 8A). The heatmap analysis revealed that the AC group exhibited higher relative abundances of Firmicutes, Chlorobi, and Cyanobacteria, while Actinobacteria, Spirochaetes, Bacteroidetes, and Chloroflexi were less abundant compared to other groups (Figure 8B). Supplementation with the AC tended to increase the relative abundance of Firmicutes while reducing that of Bacteroidetes, resulting a lower Bacteroidetes to Firmicutes ratio (0.05 ≤ *p* < 0.10) (Figure 8C–E). At the genus level, dominant genera across all groups included *Lactobacillus*, *Bacteroides*, *Faecalibacterium*, *Oscillospira*, *Enterococcus*, *Candidatus Arthromitus*, and *Parabacteroides* (Figure 8F). The heatmap analysis revealed that *Lactobacillus*, *Candidatus Arthromitus*, *Alistipes*, and *Turicibacter* were more enriched in the AC group (Figure 8G). Furthermore, compared to the CON group, dietary supplementation with the AC tended to increase the relative abundance of *Lactobacillus* in the broiler cecum (*p =* 0.065) (Figure 8H). Additionally, AC supplementation significantly reduced the relative abundances of *Bacteroides* and *Coprococcus* (*p* < 0.05) and tended to decrease the abundance of *Anaeroplasma* (*p =* 0.093) (Figure 8I–K).

## 4. Discussion

Lipid oxidation leads to the production of free fatty acids, with the acid value (AV) serving as an indicator of their concentration during the oxidation process [22]. Additionally, lipid oxidation is associated with the formation of hydroperoxides, which can be quantified using the peroxide value (POV) [23]. Malondialdehyde (MDA), a final product of lipid oxidation, is widely recognized as a marker for lipid deterioration [24]. In this study, the addition of the AC significantly reduced the AV, POV, and MDA levels during storage, demonstrating greater efficacy than EQ after six weeks.

The addition of BHT [25,26] or TBHQ [27] alone has a limited impact on broiler performance. Another study found that citric acid enhances broiler body weight gain; however, its effective dosage is as high as 5000 g/ton [28], and it remains unclear whether lower dosages would yield similar effects. Recent studies show that these antioxidants can improve animal growth performance when incorporated in a complex form [29,30]. In this study, the inclusion of the AC in the diet significantly reduced the feed conversion ratio compared to both the CON and EQ groups, consistent with findings from previous studies. The improved performance may be attributed to a reduction in feed oxidation, enhanced liver and intestinal antioxidant capacity, and improved intestinal health [31,32].

The liver serves as the primary site for regulating oxidative stress, maintaining redox balance via antioxidant enzymes [33]. Superoxide dismutase (SOD) promotes the degradation of superoxide anions and acts as the first line of defense in antioxidant systems. Additionally, total antioxidant capacity (T-AOC) can be utilized to assess the overall activity of antioxidant enzymes and substances present in tissues [34]. This study showed that dietary AC supplementation significantly enhances T-AOC in broiler livers, likely due to the significant increase in SOD levels. These findings align with the results of some previous studies that supplemented diets with BHT or citric acid alone [12,28]. Moreover, we did not observe any significant changes in the levels of catalase (CAT), glutathione peroxidase (GSH-Px), glutathione (GSH), or MDA. This may be because oxidative stress originating from the intestine has been partially neutralized during metabolic processes. Therefore, it is essential to investigate the status of intestinal health to gain a more comprehensive understanding of the effects of antioxidants.

The intestine, a vital organ for nutrient digestion and absorption, is directly exposed to external factors and is particularly susceptible to oxidative damage from dietary and environmental sources [35]. NRF2, a transcriptional activator involved in redox regulation, directly regulates the expression of numerous antioxidant enzymes, including catalase (CAT), glutathione peroxidase 7 (GPX7), heme oxygenase-1 (HO-1), and NAD(P)H quinone dehydrogenase 1 (NQO1) [36]. Both CAT and GPX7 play crucial roles in eliminating peroxides within the body. In addition to its protective effects against oxidative damage, HO-1 has recently been recognized for its significant anti-inflammatory properties [37]. NQO1 contributes to the prevention of oxidative stress by detoxifying superoxide and facilitating the antioxidant forms of physiological substrates, including ubiquinone and vitamin E quinone [38]. This study demonstrates that dietary supplementation with the AC or EQ effectively enhances the expression of antioxidant protection genes, including CAT, HO-1, GPX7, and NQO1. Additionally, Kelch-like ECH-associated protein 1 (KEAP1) acts as a negative regulator of NRF2; its downregulation leads to increased translocation of NRF2 into the nucleus, subsequently inducing the transcription of genes responsible for antioxidant protection [39]. Notably, EQ appears to elevate the expression of its downstream genes by upregulating the relative levels of NRF2 protein, while AC promotes greater nuclear entry of NRF2 through the downregulation of KEAP1, thereby facilitating antioxidant protection within the organism. Further analysis is required to elucidate the detailed mechanisms involved.

Villus height (VH) and the villus-to-crypt ratio (VCR) in the intestine are positively correlated with digestive and absorptive capacities [40]. This study found that AC supplementation significantly increased duodenal VCR and jejunal VH compared to the CON group. Additionally, the AC improved duodenal VCR, jejunal VH, crypt depth (CD), and VCR compared to the EQ group. Citric acid, a key component of AC, has been shown to improve intestinal villi morphology [41]. Broilers in the AC group exhibited more mature intestinal villi morphology, contributing to better nutrient digestion and absorption and ultimately improved growth performance. Compared to both the CON and EQ groups, AC supplementation also significantly lowered serum levels of diamine oxidase (DAO) and D-Lactate, markers of intestinal permeability, suggesting enhanced intestinal barrier function. Intestinal tight junction (TJ) proteins, including Occludin and ZO-1, are crucial components of the intestinal barrier [42]. Damage to TJs can increase intestinal permeability, allowing intraluminal antigens or bacteria to access the mucosa [43]. Additionally, MUC2 is an intestinal mucin secreted by goblet cells, forming a protective mucus layer on the intestinal epithelial surface, resisting microbial invasion [44]. This study found that the addition of the AC significantly increased the expression of Occludin, ZO-1, and MUC2 in the intestines of broilers, which may elucidate the observed improvement in intestinal barrier permeability.

Intestinal inflammation frequently damages intestinal cells [45], resulting in maldigestion and malabsorption of nutrients, which subsequently impairs host growth and development [46]. This study found that EQ increased the gene expression of the inflammatory markers *IL-1β*, *IL-6*, and *IFN-γ*, while the AC reduced *TNF-α* expression. These results align with previous findings on the anti-inflammatory effects of BHT and citric acid [12,41]. Toll-like receptor 4 (TLR4), activated by lipopolysaccharide (LPS) from Gram-negative bacteria, induces inflammatory cytokine production [47]. In the EQ group, high TLR4 expression may have triggered *IL-1β*, *IL-6*, and *IFN-γ* expression. Conversely, a more intact intestinal barrier in the AC group may have reduced TLR4 activation, thereby lowering inflammation. NF-κB, a crucial regulator of inflammation, is essential for maintaining epithelial integrity and intestinal immune homeostasis. A deficiency in NF-κB results in the apoptosis of intestinal epithelial cells, leading to tissue damage and bacterial translocation into the mucosa [48]. NF-κB p50 is a key member of the NF-κB family [49]. The precursor of p50, NF-κB p105, exerts a negative regulatory function on p50 [50]. Mice deficient in NF-κB p105 but expressing mature NF-κB p50 spontaneously develop intestinal inflammation [51]. IκB is a protein that inhibits the overactivation of NF-κB by maintaining it in an inactive state within the cytoplasm, thereby preventing its binding to DNA [52]. The current study found that the addition of EQ or the AC to the diet upregulated NF-κB p50 and its inhibitory factors NF-κB p105 and IκB concurrently, which may enhance the regulatory capacity of immune homeostasis.

The large number of microorganisms in the intestine form a distinct microecological system with the host, participating in the host’s metabolism, growth, and immunity [53]. Studies have demonstrated that antioxidants can influence host health by altering the composition of intestinal microorganisms [54,55]. To investigate the effect of the AC on the intestinal microbiota of broilers, we conducted an analysis of microbial differences among each group using 16S rDNA sequencing. The results indicated that the AC group shared fewer amplicon sequence variants (ASVs) with the other groups, exhibiting a separation tendency in its bacterial flora characteristics from those of other groups. The AC group showed a higher abundance of Firmicutes and a lower abundance of Bacteroidetes compared to the CON group, resulting in a decreased ratio of Bacteroidetes to Firmicutes. Firmicutes, primarily Gram-positive bacteria, play a crucial role in host nutrition and metabolism through the synthesis of short-chain fatty acids, particularly butyrate. Butyrate serves as an energy source for the development of intestinal epithelial cells, strengthens the intestinal barrier, and possesses anti-inflammatory and antibacterial properties [56,57]. The growth performance of animals is positively correlated with the abundance of Firmicutes and negatively correlated with the ratio of Bacteroidetes to Firmicutes [58,59]. The increased abundance of Firmicutes observed in the broilers of the AC group may enhance their growth performance. In addition, Bacteroidetes preferentially consumes proteins in mucin to sustain its survival [60]. The reduced abundance of Bacteroidetes in the AC group may have contributed to an increased richness of mucin, thereby enhancing the integrity of the intestinal barrier. Moreover, the AC group was enriched in *Lactobacillus*, *Candidatus Arthromitus, Alistipes,* and *Turicibacter* at the genus level. *Candidatus Arthromitus* plays a critical role in the maturation of the host gut immune barrier, inducing both innate and adaptive immune responses [61,62]. Furthermore, higher-performing flocks exhibit a greater proportion of *Candidatus Arthromitus*, suggesting that these bacteria may enhance intestinal health and protect commercial turkeys from pathogens that adversely affect production parameters [63]. Additionally, *Turicibacter* has been shown to enhance host lipid metabolism by modifying bile acids [64]. *Alistipes* members are important producers of short-chain fatty acids (SCFAs), recognized as growth promoters for broilers [65,66]. Moreover, some members of *Alistipes* exhibit anti-inflammatory and hyperuricemia-relieving properties [67,68]. A comparative analysis between the AC group and the CON group revealed that the relative abundance of *Lactobacillus* in the broilers of the AC group increased, while the relative abundance of *Bacteroides*, *Coprococcus*, and *Anaeroplasma* decreased. *Lactobacilli*, belonging to the phylum Firmicutes, are the most prevalent probiotics that produce lactic acid, acetic acid, and propionic acid, which can lower intestinal pH and inhibit the growth of various pathogenic bacteria, thereby maintaining the balance of intestinal flora [69]. Incorporating Lactobacillus into broiler diets has been shown to enhance the intestinal barrier of broilers, regulate intestinal microbiota, and provide immune protection [70,71]. *Coprococcus* has been linked to bacterial overgrowth in the small intestine [72], while *Bacteroides*, a predominant genus in Bacteroidetes, adversely affects broiler growth performance. *Anaeroplasma* is an opportunistic pathogen whose abundance significantly increases in mice with inflammation-driven colon cancer [73,74]. This study suggests that incorporating the AC into the diet may increase the abundance of beneficial gut microbes while reducing the abundance of harmful bacteria and those negatively associated with growth performance, thereby exerting a favorable influence on the host.

## 5. Conclusions

**Figure 9:**
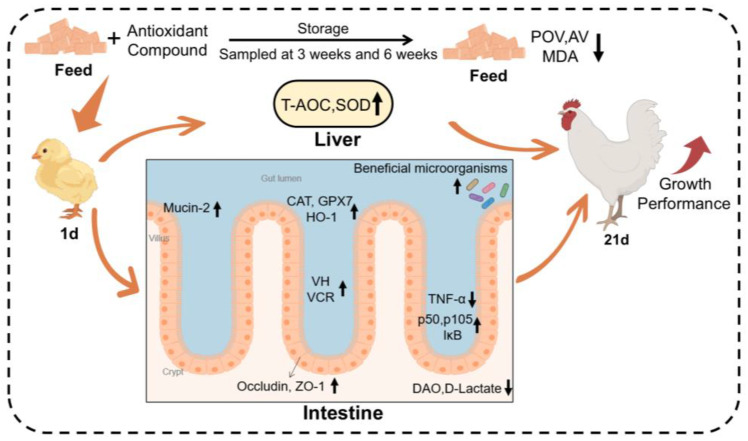
Graphic summary of the effects of the antioxidant compound on feed antioxidant protection and broiler health. Antioxidant compound: including 18% butylated hydroxytoluene, 3% citric acid, and 1% tertiary butylhydroquinone.

This study demonstrates that a novel antioxidant compound, composed of 18% BHT, 3% citric acid, and 1% TBHQ, effectively delays feed oxidation and improves broiler growth performance by enhancing antioxidant capacity and promoting intestinal health (Figure 9). Moreover, this compound exhibites superior effects compared to ethoxyquin in both in vivo and in vitro experiments.

## Figures and Tables

**Table 1:** Ingredients and nutrient composition of the basal diet.

Ingredients	%	Nutrient Parameters ^3^	Nutrient Level
Corn	52.35	Metabolizable energy, Kcal/kg	2950
Soybean meal	38.71	Crude protein, %	22.0
Corn gluten meal	1.31	Lys, %	1.31
Soybean oil	3.06	Met, %	0.48
Dicalcium phosphate	1.79	Met + Cys, %	0.96
Limestone	1.30	Thr, %	0.86
Sodium chloride	0.35	Val, %	0.89
Choline chloride (50%)	0.20	Calcium, %	1.02
L-Lysine Hydrochloride (98.5%)	0.26	Non-phytate phosphorus, %	0.39
DL-Methionine (98%)	0.31		
L-Threonine (98.5%)	0.05		
L-Isoleucine (90%)	0.03		
L-Arginine (98%)	0.02		
Phytase (10,000 U/g)	0.03		
Mineral premix ^1^	0.20		
Vitamin premix ^2^	0.03		
Total	100		

^1^ The mineral premix provided the following per kg of diets: Cu, 16 mg; Zn, 110 mg; Fe, 80 mg; Mn, 120 mg; Se, 0.30 mg; I, 1.50 mg. ^2^ The vitamin premix provided the following per kg of diets: vitamin A, 15,000 IU; vitamin D3, 3600 IU; vitamin E, 30 IU; vitamin K3, 3.00 mg; vitamin B2, 9.60 mg; vitamin B12, 0.03 mg; biotin, 0.15 mg; folic acid, 1.50 mg; pantothenic acid, 13.80 mg; nicotinic acid, 45 mg. ^3^ Nutrient and amino acid composition of cereals and protein sources were analyzed using near-infrared spectroscopy prior to feed formulation. The nutrient composition of the basal diet is based on calculated values.

**Table 2:** Primer sequences used for quantitative real-time PCR analysis.

Gene ^1^	Primer Sequences (5′-3′) ^2^	Accession Number
*β-actin*	F: TTGTTGACAATGGCTCCGGT	NM_205518.1
	R: TCTGGGCTTCATCACCAACG	
*IFN-γ*	F: CTCGCAACCTTCACCTCACCATC	NM_205149.1
	R: CAGGAACCAGGCACGAGCTTG	
*IL-6*	F: GAACGTCGAGTCTCTGTGCTAC	NM_204628
	R: CACCATCTGCCGGATCGT	
*IL-1β*	F: CAGCCTCAGCGAAGAGACCTT	NM_204524
	R: ACTGTGGTGTGCTCAGAATCC	
*TNF-α*	F: CCCCTACCCTGTCCCACAA	NM_204267
	R: TGAGTACTGCGGAGGGTTCAT	
*IL-18*	F: GTGTGTGCAGTACGGCTTAG	NM_204608.1
	R: TCCACTGCCAGATTTCACCT	

^1^ Interferon γ (*IFN-γ*), interleukin 6 (*IL-6*), interleukin 1β (*IL-1β*), tumor necrosis factor-alpha (*TNF-α*), and interleukin 18 (*IL-18*). ^2^ F, forward primer; R, reverse primer.

**Table 3:** Intestinal morphology of broilers.

Item ^1^	Duodenum			Jejunum		
	VH	CD	VCR	VH	CD	VCR
CON	1413.64	214.71	6.28 ^b^	965.15 ^b^	175.31 ^b^	5.57 ^a^
EQ	1253.73	211.79	5.93 ^b^	752.79 ^c^	165.16 ^b^	4.38 ^b^
AC	1366.46	204.85	7.38 ^a^	1112.82 ^a^	203.73 ^a^	5.07 ^a^
SEM	29.265	4.838	0.159	37.593	5.567	0.147
*p*-Value	0.067	0.71	<0.001	<0.001	0.008	0.001

^1^ CON, basal diet; EQ, basal diet + 200 g/ton ethoxyquin; AC, basal diet + 200 g/ton antioxidant compound. N = 9. Abbreviations: VH, villus height; CD, crypt depth; VCR, villus height to crypt depth ratio. ^abc^ Different superscript letters indicate significant differences (*p* < 0.05).

## Data Availability

The 16S rRNA amplicon sequencing reads from this dataset have been deposited in the National Center for Biotechnology Information under BioProjectID PRJNA1158563. Additionally, the data presented in this study are available on request from the corresponding author.
